# 2-Methyl­benzimidazolium thio­cyanate–2-methyl­benzimidazole (1/1)

**DOI:** 10.1107/S1600536810031181

**Published:** 2010-08-11

**Authors:** Shayma A. Shaker, Hamid Khaledi, Hapipah Mohd Ali

**Affiliations:** a Department of Chemistry, University of Malaya, 50603 Kuala Lumpur, Malaysia

## Abstract

In the crystal structure of the title compound, C_8_H_9_N_2_^+^·SCN^−^·C_8_H_8_N_2_, the three components are linked by inter­molecular N—H⋯N and N—H⋯S hydrogen bonds into infinite chains along the *c* axis.

## Related literature

For related structures, see: Bhattacharya *et al.* (2004 ▶); Ding *et al.* (2004 ▶); Huang *et al.* (2006 ▶). For applications of benzimidazole derivatives in crystal engineering, see: Cai *et al.* (2002 ▶). For the biological properties of benzimidazole derivatives, see: Refaat (2010 ▶); Ansari & Lal (2009 ▶).
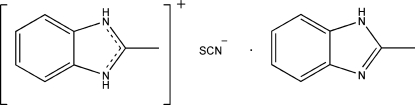


## Experimental

### 

#### Crystal data

C_8_H_9_N_2_^+^·SCN^−^·C_8_H_8_N_2_
*M* *_r_* = 323.42
Monoclinic, 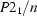

*a* = 11.0952 (7) Å
*b* = 6.9664 (4) Å
*c* = 21.4195 (13) Å
β = 100.745 (1)°
*V* = 1626.56 (17) Å^3^
*Z* = 4
Mo *K*α radiation
μ = 0.21 mm^−1^
*T* = 100 K
0.25 × 0.25 × 0.06 mm

#### Data collection

Bruker APEXII CCD diffractometer
Absorption correction: multi-scan (*SADABS*; Sheldrick, 1996 ▶) *T*_min_ = 0.950, *T*_max_ = 0.988
8812 measured reflections
3193 independent reflections
2427 reflections with *I* > 2σ(*I*)
*R*_int_ = 0.037

#### Refinement

*R*[*F* ^2^ > 2σ(*F* ^2^)] = 0.039
*wR*(*F* ^2^) = 0.093
*S* = 1.03
3193 reflections
222 parameters
3 restraints
H atoms treated by a mixture of independent and constrained refinement
Δρ_max_ = 0.20 e Å^−3^
Δρ_min_ = −0.28 e Å^−3^

### 

Data collection: *APEX2* (Bruker, 2007 ▶); cell refinement: *SAINT* (Bruker, 2007 ▶); data reduction: *SAINT*; program(s) used to solve structure: *SHELXS97* (Sheldrick, 2008 ▶); program(s) used to refine structure: *SHELXL97* (Sheldrick, 2008 ▶); molecular graphics: *X-SEED* (Barbour, 2001 ▶); software used to prepare material for publication: *SHELXL97* and *publCIF* (Westrip, 2010 ▶).

## Supplementary Material

Crystal structure: contains datablocks I, global. DOI: 10.1107/S1600536810031181/pv2314sup1.cif

Structure factors: contains datablocks I. DOI: 10.1107/S1600536810031181/pv2314Isup2.hkl

Additional supplementary materials:  crystallographic information; 3D view; checkCIF report

## Figures and Tables

**Table 1:** Hydrogen-bond geometry (Å, °)

*D*—H⋯*A*	*D*—H	H⋯*A*	*D*⋯*A*	*D*—H⋯*A*
N1—H1*N*⋯N5	0.90 (2)	1.90 (2)	2.799 (2)	176 (2)
N2—H2*N*⋯N4^i^	0.90 (2)	1.88 (2)	2.781 (2)	179 (2)
N3—H3*N*⋯S1	0.86 (2)	2.47 (2)	3.317 (2)	168 (2)

Symmetry code: (i) 
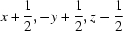
.

**Fig. 1.**
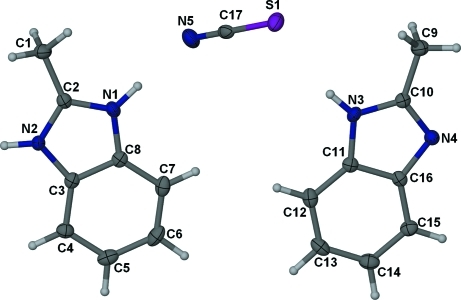
Thermal ellipsoid plot of the title compound at the 50% probability level. Hydrogen atoms are drawn as spheres of arbitrary radius.

**Fig. 2.**
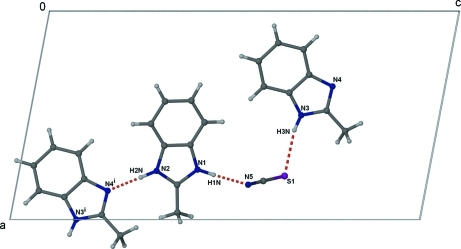
A view of the hydrogen bonding interactions as viewed down b. Symmetry code: i = x + 1/2, -y + 1/2, z - 1/2.

## supplementary crystallographic information

### Comment

Benzimidazole derivatives are biologically active compounds (Refaat, 2010; Ansari & Lal, 2009). Their applications in crystal-engineering have been reported (Cai *et al.*, 2002). The crystal structures of several compounds similar to the title compound have been publsihed (Bhattacharya *et al.*, 2004; Ding *et al.*, 2004; Huang *et al.*, 2006. In this article, the preparation and crystal structure of the title compound is presented.

The asymmetric unit of the title compound contains a 2-methylbenzimidazolium cation, a thiocyante anion and a molecule of 2-methylbenzimidazole (Fig. 1). In the crystal structure, the three moieties are linked by intramolecular N—H···N and N—H···S hydrogen bondings into infinite one-dimensional chains (Tab. 1 & Fig. 2).

### Experimental

An ethanolic solution (12 ml) of 2-methylbenzimidazole (9 mmol, 1.2 g) was added to an aqueous solution (10 ml) of FeCl_3_ (3 mmol) followed by addition of an aqueous solution (10 ml) of KSCN (9 mmol). The mixture was heated in a water bath for 15 min. The resulting precipitates were filtered off, washed with ethanol (50%) and recrystallized from ethanol whereupon the pale yellow crystals of the title compound were obtained unexpectedly.

### Refinement

The C-bound hydrogen atoms were placed at calculated positions (C—H 0.95 - 0.98 Å) and were treated as riding on their parent atoms with *U_iso_*(H) set to 1.2–1.5 *U_eq_*(C). The N-bound hydrogen atoms were located in a difference Fourier map and were refined with a distance restraint of N—H 0.88 (2) Å.

### Crystal data

**Table:**

C_8_H_9_N_2_^+^·SCN^−^·C_8_H_8_N_2_	*F*(000) = 680
*M**_r_* = 323.42	*D*_x_ = 1.321 Mg m^−^^3^
Monoclinic, *P*2_1_/*n*	Mo *K*α radiation, λ = 0.71073 Å
Hall symbol: -P 2yn	Cell parameters from 1739 reflections
*a* = 11.0952 (7) Å	θ = 2.3–25.1°
*b* = 6.9664 (4) Å	µ = 0.21 mm^−^^1^
*c* = 21.4195 (13) Å	*T* = 100 K
β = 100.745 (1)°	Plate, yellow
*V* = 1626.56 (17) Å^3^	0.25 × 0.25 × 0.06 mm
*Z* = 4	

### Data collection

**Table:**

Bruker APEXII CCD diffractometer	3193 independent reflections
Radiation source: fine-focus sealed tube	2427 reflections with *I* > 2σ(*I*)
graphite	*R*_int_ = 0.037
φ and ω scans	θ_max_ = 26.0°, θ_min_ = 1.9°
Absorption correction: multi-scan (*SADABS*; Sheldrick, 1996)	*h* = −13→13
*T*_min_ = 0.950, *T*_max_ = 0.988	*k* = −7→8
8812 measured reflections	*l* = −26→26

### Refinement

**Table:**

Refinement on *F*^2^	Primary atom site location: structure-invariant direct methods
Least-squares matrix: full	Secondary atom site location: difference Fourier map
*R*[*F*^2^ > 2σ(*F*^2^)] = 0.039	Hydrogen site location: inferred from neighbouring sites
*wR*(*F*^2^) = 0.093	H atoms treated by a mixture of independent and constrained refinement
*S* = 1.03	*w* = 1/[σ^2^(*F*_o_^2^) + (0.035*P*)^2^ + 0.6349*P*] where *P* = (*F*_o_^2^ + 2*F*_c_^2^)/3
3193 reflections	(Δ/σ)_max_ < 0.001
222 parameters	Δρ_max_ = 0.20 e Å^−^^3^
3 restraints	Δρ_min_ = −0.28 e Å^−^^3^

### Special details

**Table:**

Geometry. All e.s.d.'s (except the e.s.d. in the dihedral angle between two l.s. planes) are estimated using the full covariance matrix. The cell e.s.d.'s are taken into account individually in the estimation of e.s.d.'s in distances, angles and torsion angles; correlations between e.s.d.'s in cell parameters are only used when they are defined by crystal symmetry. An approximate (isotropic) treatment of cell e.s.d.'s is used for estimating e.s.d.'s involving l.s. planes.
Refinement. Refinement of *F*^2^ against ALL reflections. The weighted *R*-factor *wR* and goodness of fit *S* are based on *F*^2^, conventional *R*-factors *R* are based on *F*, with *F* set to zero for negative *F*^2^. The threshold expression of *F*^2^ > σ(*F*^2^) is used only for calculating *R*-factors(gt) *etc*. and is not relevant to the choice of reflections for refinement. *R*-factors based on *F*^2^ are statistically about twice as large as those based on *F*, and *R*- factors based on ALL data will be even larger.

### Fractional atomic coordinates and isotropic or equivalent isotropic displacement parameters (Å^2^)

**Table:**

	*x*	*y*	*z*	*U*_iso_*/*U*_eq_	
N1	0.76009 (14)	0.1676 (2)	0.43464 (7)	0.0183 (4)	
H1N	0.7853 (18)	0.196 (3)	0.4761 (8)	0.033 (6)*	
N2	0.76652 (13)	0.1089 (2)	0.33548 (7)	0.0169 (3)	
H2N	0.7976 (19)	0.090 (3)	0.2999 (8)	0.038 (7)*	
C1	0.96598 (16)	0.1988 (3)	0.40543 (9)	0.0233 (4)	
H1A	1.0105	0.0784	0.4165	0.035*	
H1B	0.9851	0.2889	0.4410	0.035*	
H1C	0.9908	0.2546	0.3678	0.035*	
C2	0.83267 (16)	0.1602 (3)	0.39179 (8)	0.0174 (4)	
C3	0.64561 (16)	0.0800 (3)	0.34260 (9)	0.0170 (4)	
C4	0.54091 (16)	0.0242 (3)	0.29996 (9)	0.0198 (4)	
H4	0.5429	−0.0024	0.2567	0.024*	
C5	0.43398 (17)	0.0096 (3)	0.32393 (10)	0.0244 (5)	
H5	0.3608	−0.0290	0.2963	0.029*	
C6	0.43008 (17)	0.0498 (3)	0.38747 (10)	0.0262 (5)	
H6	0.3545	0.0381	0.4019	0.031*	
C7	0.53387 (17)	0.1064 (3)	0.42968 (10)	0.0227 (4)	
H7	0.5317	0.1352	0.4728	0.027*	
C8	0.64148 (16)	0.1190 (3)	0.40569 (9)	0.0173 (4)	
N3	0.49936 (14)	0.4693 (2)	0.66246 (7)	0.0179 (3)	
H3N	0.5709 (14)	0.483 (3)	0.6527 (9)	0.022 (5)*	
N4	0.36143 (13)	0.4455 (2)	0.72545 (7)	0.0175 (3)	
C9	0.57509 (16)	0.5348 (3)	0.77709 (9)	0.0236 (4)	
H9A	0.5377	0.5843	0.8119	0.035*	
H9B	0.6283	0.6334	0.7640	0.035*	
H9C	0.6239	0.4205	0.7915	0.035*	
C10	0.47689 (16)	0.4834 (3)	0.72223 (9)	0.0174 (4)	
C11	0.39134 (16)	0.4178 (3)	0.62290 (9)	0.0173 (4)	
C12	0.36142 (17)	0.3839 (3)	0.55802 (9)	0.0212 (4)	
H12	0.4209	0.3944	0.5315	0.025*	
C13	0.24092 (18)	0.3343 (3)	0.53365 (9)	0.0235 (4)	
H13	0.2171	0.3092	0.4895	0.028*	
C14	0.15347 (18)	0.3204 (3)	0.57299 (9)	0.0239 (4)	
H14	0.0715	0.2865	0.5549	0.029*	
C15	0.18384 (16)	0.3548 (3)	0.63752 (9)	0.0204 (4)	
H15	0.1240	0.3455	0.6638	0.024*	
C16	0.30494 (16)	0.4037 (3)	0.66294 (8)	0.0167 (4)	
S1	0.79070 (4)	0.52630 (8)	0.64995 (2)	0.02656 (15)	
N5	0.83250 (16)	0.2375 (3)	0.56491 (8)	0.0283 (4)	
C17	0.81466 (16)	0.3567 (3)	0.60007 (9)	0.0208 (4)	

### Atomic displacement parameters (Å^2^)

**Table:**

	*U*^11^	*U*^22^	*U*^33^	*U*^12^	*U*^13^	*U*^23^
N1	0.0217 (8)	0.0189 (9)	0.0143 (8)	0.0006 (7)	0.0030 (6)	−0.0004 (7)
N2	0.0162 (8)	0.0193 (9)	0.0156 (8)	0.0004 (6)	0.0041 (6)	−0.0006 (7)
C1	0.0184 (10)	0.0266 (12)	0.0237 (10)	0.0000 (8)	0.0006 (8)	−0.0034 (9)
C2	0.0200 (9)	0.0152 (10)	0.0169 (9)	0.0024 (7)	0.0029 (7)	0.0009 (8)
C3	0.0186 (9)	0.0132 (10)	0.0198 (10)	0.0019 (7)	0.0051 (7)	0.0022 (8)
C4	0.0197 (9)	0.0175 (10)	0.0210 (10)	0.0005 (8)	0.0008 (7)	0.0007 (9)
C5	0.0181 (9)	0.0196 (11)	0.0343 (12)	−0.0004 (8)	0.0016 (8)	0.0046 (9)
C6	0.0213 (10)	0.0226 (11)	0.0380 (12)	0.0022 (8)	0.0145 (9)	0.0068 (10)
C7	0.0283 (11)	0.0180 (11)	0.0248 (10)	0.0030 (8)	0.0125 (8)	0.0040 (9)
C8	0.0197 (9)	0.0115 (10)	0.0207 (10)	0.0015 (7)	0.0043 (7)	0.0028 (8)
N3	0.0143 (8)	0.0202 (9)	0.0205 (8)	0.0002 (7)	0.0063 (6)	0.0005 (7)
N4	0.0178 (8)	0.0181 (9)	0.0165 (8)	−0.0002 (6)	0.0031 (6)	0.0013 (7)
C9	0.0197 (10)	0.0262 (11)	0.0242 (10)	−0.0011 (8)	0.0021 (8)	−0.0001 (9)
C10	0.0180 (9)	0.0149 (10)	0.0193 (9)	0.0001 (7)	0.0032 (7)	−0.0002 (8)
C11	0.0192 (9)	0.0131 (10)	0.0193 (9)	0.0011 (7)	0.0027 (7)	0.0009 (8)
C12	0.0291 (10)	0.0159 (10)	0.0201 (10)	0.0016 (8)	0.0083 (8)	0.0022 (8)
C13	0.0328 (11)	0.0177 (11)	0.0177 (10)	−0.0005 (9)	−0.0011 (8)	0.0000 (9)
C14	0.0230 (10)	0.0202 (11)	0.0258 (11)	−0.0027 (8)	−0.0018 (8)	0.0027 (9)
C15	0.0182 (9)	0.0188 (11)	0.0240 (10)	−0.0017 (8)	0.0032 (8)	0.0029 (9)
C16	0.0213 (9)	0.0126 (9)	0.0159 (9)	0.0023 (7)	0.0025 (7)	0.0027 (8)
S1	0.0226 (3)	0.0293 (3)	0.0296 (3)	−0.0040 (2)	0.0095 (2)	−0.0066 (2)
N5	0.0336 (10)	0.0324 (11)	0.0184 (9)	0.0026 (8)	0.0033 (7)	−0.0006 (8)
C17	0.0166 (9)	0.0285 (12)	0.0165 (9)	−0.0007 (8)	0.0010 (7)	0.0060 (9)

### Geometric parameters (Å, °)

**Table:**

N1—C2	1.330 (2)	N3—C11	1.380 (2)
N1—C8	1.388 (2)	N3—H3N	0.862 (15)
N1—H1N	0.901 (15)	N4—C10	1.322 (2)
N2—C2	1.338 (2)	N4—C16	1.399 (2)
N2—C3	1.393 (2)	C9—C10	1.489 (2)
N2—H2N	0.902 (15)	C9—H9A	0.9800
C1—C2	1.478 (2)	C9—H9B	0.9800
C1—H1A	0.9800	C9—H9C	0.9800
C1—H1B	0.9800	C11—C12	1.387 (3)
C1—H1C	0.9800	C11—C16	1.404 (3)
C3—C8	1.387 (2)	C12—C13	1.385 (3)
C3—C4	1.392 (2)	C12—H12	0.9500
C4—C5	1.381 (3)	C13—C14	1.402 (3)
C4—H4	0.9500	C13—H13	0.9500
C5—C6	1.398 (3)	C14—C15	1.381 (3)
C5—H5	0.9500	C14—H14	0.9500
C6—C7	1.382 (3)	C15—C16	1.395 (2)
C6—H6	0.9500	C15—H15	0.9500
C7—C8	1.388 (3)	S1—C17	1.647 (2)
C7—H7	0.9500	N5—C17	1.163 (2)
N3—C10	1.353 (2)		
			
C2—N1—C8	109.14 (15)	C10—N3—C11	107.93 (15)
C2—N1—H1N	124.9 (13)	C10—N3—H3N	124.1 (13)
C8—N1—H1N	125.9 (13)	C11—N3—H3N	127.8 (13)
C2—N2—C3	108.51 (15)	C10—N4—C16	104.90 (15)
C2—N2—H2N	124.6 (14)	C10—C9—H9A	109.5
C3—N2—H2N	126.8 (14)	C10—C9—H9B	109.5
C2—C1—H1A	109.5	H9A—C9—H9B	109.5
C2—C1—H1B	109.5	C10—C9—H9C	109.5
H1A—C1—H1B	109.5	H9A—C9—H9C	109.5
C2—C1—H1C	109.5	H9B—C9—H9C	109.5
H1A—C1—H1C	109.5	N4—C10—N3	112.71 (15)
H1B—C1—H1C	109.5	N4—C10—C9	125.46 (17)
N1—C2—N2	109.35 (15)	N3—C10—C9	121.82 (16)
N1—C2—C1	124.67 (16)	N3—C11—C12	132.64 (17)
N2—C2—C1	125.96 (17)	N3—C11—C16	104.88 (16)
C8—C3—C4	121.23 (17)	C12—C11—C16	122.48 (17)
C8—C3—N2	106.61 (15)	C13—C12—C11	116.98 (17)
C4—C3—N2	132.17 (17)	C13—C12—H12	121.5
C5—C4—C3	116.49 (18)	C11—C12—H12	121.5
C5—C4—H4	121.8	C12—C13—C14	121.20 (18)
C3—C4—H4	121.8	C12—C13—H13	119.4
C4—C5—C6	122.11 (18)	C14—C13—H13	119.4
C4—C5—H5	118.9	C15—C14—C13	121.49 (18)
C6—C5—H5	118.9	C15—C14—H14	119.3
C7—C6—C5	121.38 (18)	C13—C14—H14	119.3
C7—C6—H6	119.3	C14—C15—C16	118.08 (17)
C5—C6—H6	119.3	C14—C15—H15	121.0
C6—C7—C8	116.43 (18)	C16—C15—H15	121.0
C6—C7—H7	121.8	C15—C16—N4	130.66 (17)
C8—C7—H7	121.8	C15—C16—C11	119.76 (17)
C3—C8—C7	122.36 (17)	N4—C16—C11	109.58 (15)
C3—C8—N1	106.39 (15)	N5—C17—S1	179.47 (18)
C7—C8—N1	131.25 (18)		

### Hydrogen-bond geometry (Å, °)

**Table:**

*D*—H···*A*	*D*—H	H···*A*	*D*···*A*	*D*—H···*A*
N1—H1N···N5	0.90 (2)	1.90 (2)	2.799 (2)	176 (2)
N2—H2N···N4^i^	0.90 (2)	1.88 (2)	2.781 (2)	179 (2)
N3—H3N···S1	0.86 (2)	2.47 (2)	3.317 (2)	168 (2)

Symmetry codes: (i) *x*+1/2, −*y*+1/2, *z*−1/2.
